# Supplementary material for: Fine-tuning foundation models of materials interatomic potentials with frozen transfer learning
Source: NPJ Comput Mater. 2025 Jul 18;11(1):237. doi: 10.1038/s41524-025-01727-x (PMC12274129; doi:10.1038/s41524-025-01727-x)
Supplement: Supplementary file 1 — Supplementary information [file 41524_2025_1727_MOESM1_ESM.pdf]

# Supplementary Information to "Fine-tuning foundation models of materials interatomic potentials with frozen transfer learning"

Mariia Radova,[1,2] Wojciech G. Stark,[1] Connor Allen,[2,3] Reinhard J. Maurer,[1,2,*] and Albert P. Bartók[2,3,†]

[1] *Department of Chemistry, University of Warwick, Gibbet Hill Road, Coventry CV4 7AL, United Kingdom*

[2] *Department of Physics, University of Warwick, Gibbet Hill Road, Coventry CV4 7AL, United Kingdom*

[3] *Warwick Centre for Predictive Modelling, School of Engineering, University of Warwick, Gibbet Hill Road, Coventry CV4 7AL, United Kingdom*

## S1. FINE TUNING OF FOUNDATION MODELS WITH DELTA LEARNING

Delta learning has been previously used in the context of machine learning interatomic potentials [1] with a SchNet [2] model. This technique allows to learn the difference between a baseline (in this case, MACE-MP) method and a high accuracy method (DFT). The energies and forces of the relevant structures are replaced with the corresponding differences between their values predicted by the foundation models and the reference values, later referred to as "deltas". The delta models are small models trained on the deltas and their predictions are then added to the predictions of the baseline models:

$$\epsilon_{\mathrm{pred}} = \epsilon_{\Delta} + \epsilon_{\mathrm{MP}} \tag{1}$$

$$\epsilon_{\Delta} = \epsilon_{\mathrm{MP}} - \epsilon_{\mathrm{DFT}} \tag{2}$$

Delta learning is meant to be a cheap and efficient way of fine-tuning a machine learning model, with the assumption that the deltas can be fit, and the fitting function is simple enough so that smaller models with less number of parameters can learn the deltas.

## S2. SUPPLEMENTARY FIGURES

* r.maurer@warwick.ac.uk

† Albert.Bartok-Partay@warwick.ac.uk

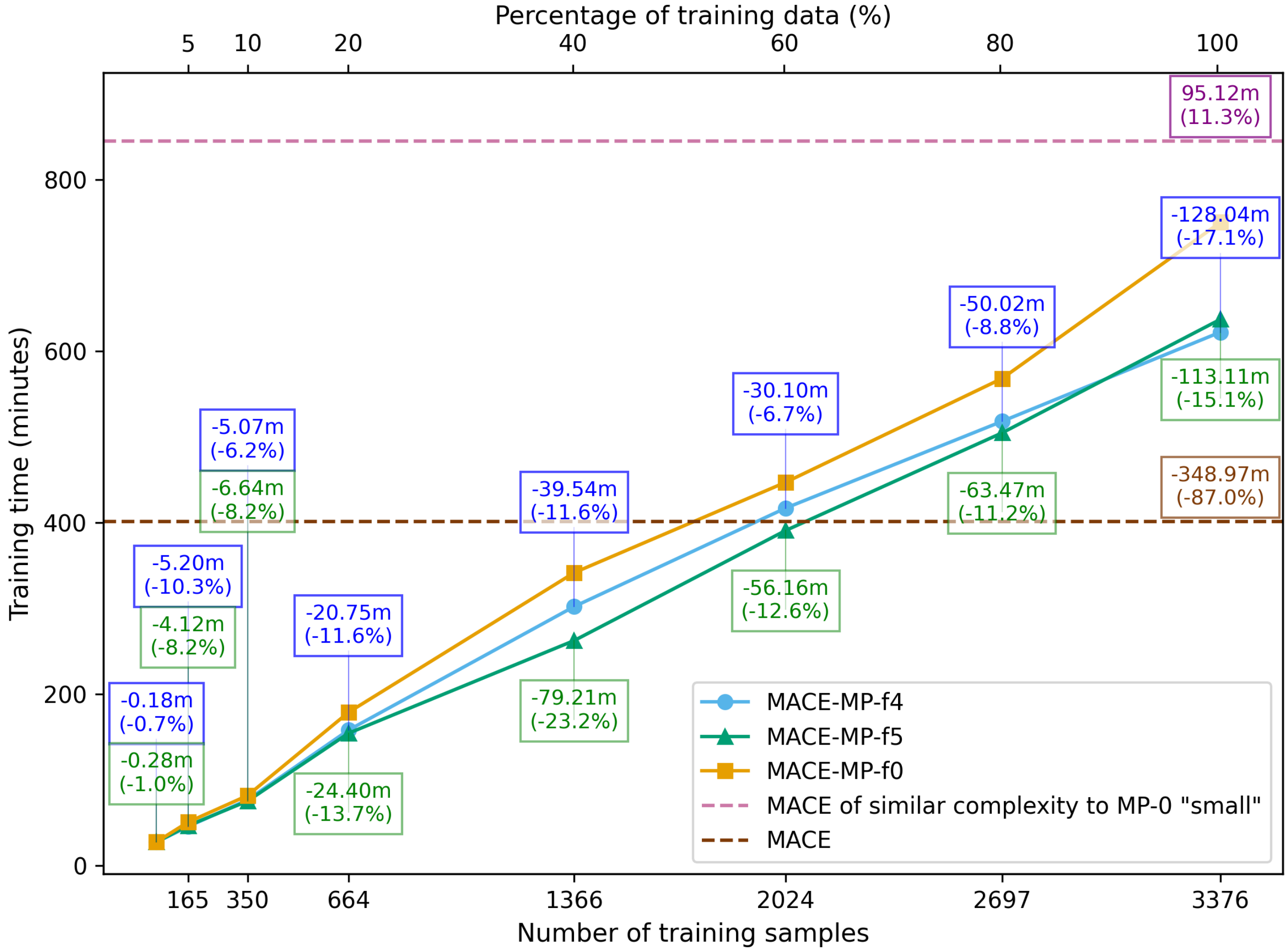


FIG. S1. **Effective training time of Cu-H models.** Training times are shown for the MACE-MP-f4 (blue circles), MACE-MP-f5 (green triangles) and MACE-MP-f0 (yellow squares) transfer models, and MACE optimised (brown dashed line) and MACE of the similar size and complexity to MACE-MP-0 "small" (purple dashed line). The speedup is calculated relative to the MACE-MP-f0 models. This plot presents the effective training time for the Cu-H system, which is influenced by two primary factors: the acceleration of epoch completion due to parameter freezing and the time required to obtain the "best model" saved by MACE, given that all models are trained for the same number of epochs. Consequently, fluctuations in percentage values are expected. However, these variations should provide a representative estimate of the computational time savings achievable by the user.

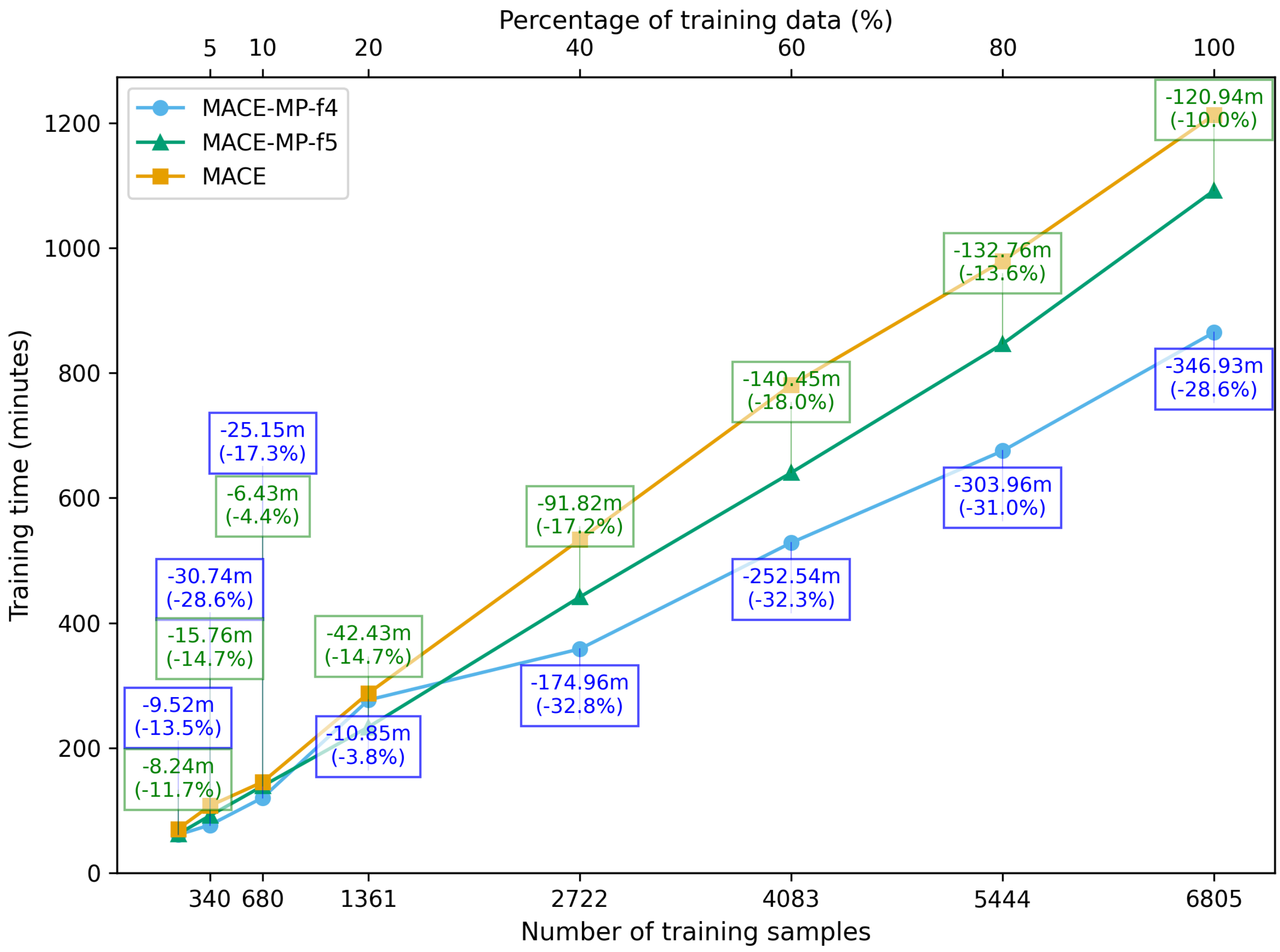


FIG. S2. **Effective training time of Ti-Al-V models.** Training times are shown for the MACE-MP-f4 (blue circles), MACE-MP-f5 (green triangles) and MACE from-scratch (yellow squares). Here, the speedup is measured relative to the from-scratch MACE model of similar size to MACE-MP-0 "small". Notably, for the Ti-Al-V system, despite backpropagation being faster in the MACE-MP-f5 setting, convergence to the "best model" occurred more quickly with the MACE-MP-f4 setting. This behavior contrasts with the Cu-H system, where such a trend was not observed.

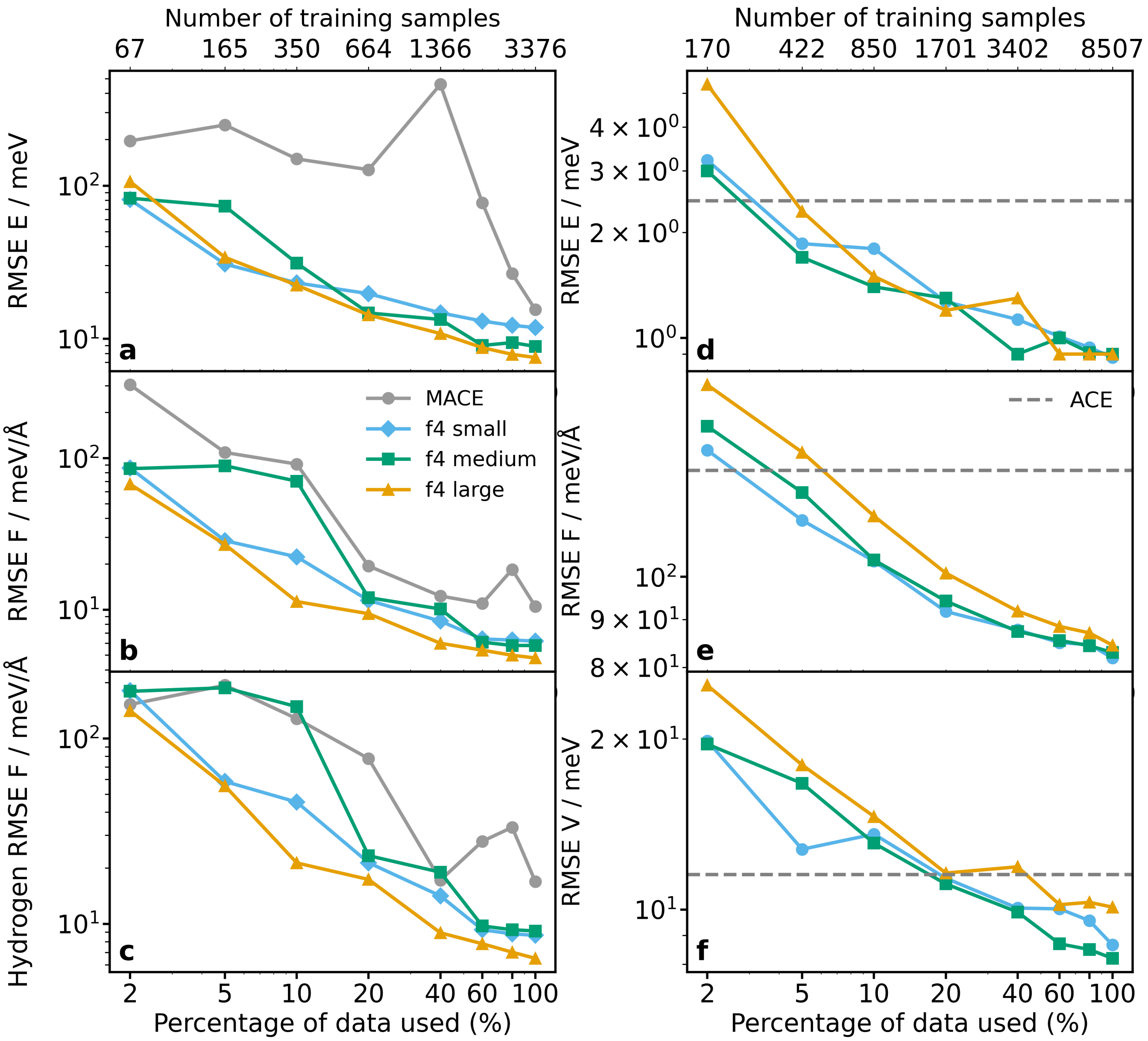


FIG. S3. **Transfer learning curves for Hydrogen on Copper surface and the Ti-Al-V systems.** Panels (a), (b), (c) refer to the Cu-$H_2$ system, and panels (d), (e), (f) refer to the Ti-Al-V system. These transfer models were trained on MACE-MP “small” (blue diamonds), “medium” (green squares), and “large” (yellow triangles) foundation models. The gray circles mark the learning curve of the from-scratch MACE model. The gray dashed line marks the errors of the custom ACE model. The points correspond to the percentages of the full dataset, namely 2, 5, 10, 20, 40, 60, 80 and 100 %.

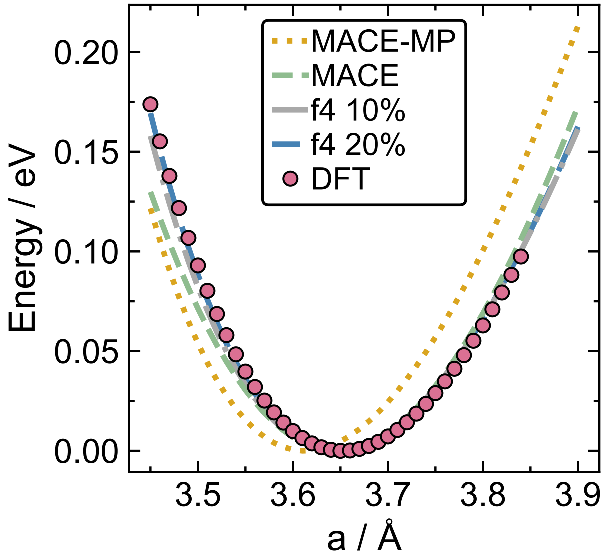


FIG. S4. **Relative potential energies of the Cu atoms for a set of lattice constants**. Energies were calculated using DFT (red circles) and all the MLIPs included in our study, namely, MACE (green, shortly-dashed line), MACE-MP (yellow, dotted line), MACE-MP-f4 10% (f4 10%) (gray, “dash-dot” line), and MACE-MP-f4 20% (f4 20%) (blue, dashed line).

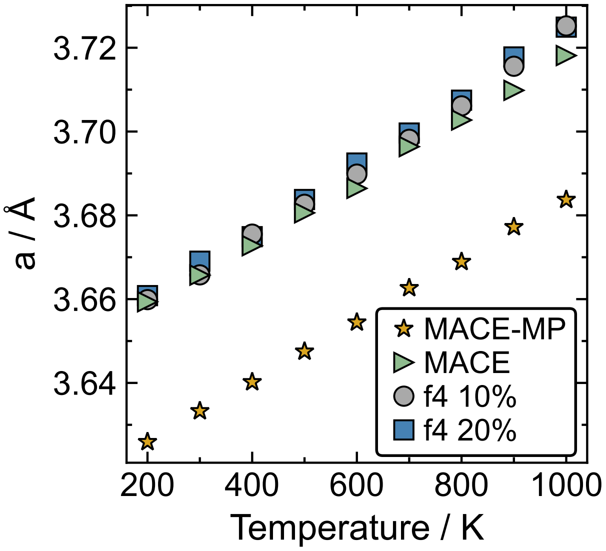


FIG. S5. **Relation between the lattice constant of the Cu slab and temperature.**. The lattice constants were extracted from NPT simulations at 9 temperatures ranging from 200 and 1000 K, using MACE-MP (yellow stars), from-scratch MACE (green triangles), MACE-MP-f4 10% (grey circles), and MACE-MP-f4 20% (blue squares) models.

| | ACE | MACE-MP-f 2% | MACE-MP-f 5% | MACE-MP-f 10% | MACE-MP-f 20% | MACE-MP-f 40% | MACE-MP-f 60% | MACE-MP-f 80% | MACE-MP-f 100% |
|---|---|---|---|---|---|---|---|---|---|
| E_train (eV) | 0.0037500 | 0.012090 | 0.0073600 | 0.0088000 | 0.0049100 | 0.0039000 | 0.0036400 | 0.0033900 | 0.0032200 |
| F_train (eV/AA) | 0.14928 | 0.23799 | 0.21017 | 0.31895 | 0.17202 | 0.13949 | 0.12761 | 0.15240 | 0.11497 |
| V_train (eV) | 0.024130 | 0.093680 | 0.062720 | 0.070600 | 0.045690 | 0.040410 | 0.038720 | 0.040390 | 0.036210 |
| E_validation (eV) | 0.0024700 | 0.0073900 | 0.0038000 | 0.0025300 | 0.0015000 | 0.0012100 | 0.0016700 | 0.0015400 | 0.0014100 |
| F_validation (eV/AA) | 0.12993 | 0.13876 | 0.12826 | 0.11805 | 0.11252 | 0.10442 | 0.10349 | 0.10073 | 0.099360 |
| V_validation (eV) | 0.011540 | 0.050300 | 0.038980 | 0.023640 | 0.019770 | 0.014480 | 0.013630 | 0.013880 | 0.012370 |
| hcp_Ti7AlV (THz) | 0.25810 | 0.36801 | 0.23965 | 0.26235 | 0.29152 | 0.36497 | 0.31178 | 0.31370 | 0.30950 |
| hcp_Ti7AlV deltaF 0K (eV) | 0.00038000 | 0.0010000 | 0.00024000 | 0.00043000 | 0.00054000 | 0.0013400 | 0.0010400 | 0.00093000 | 0.0010800 |
| hcp_Ti7AlV deltaF 2000K (eV) | 0.025320 | 0.041270 | 0.015840 | 0.027410 | 0.032170 | 0.044660 | 0.039480 | 0.035760 | 0.039430 |
| bcc_Ti7AlV (THz) | 0.64197 | 0.79593 | 0.65898 | 0.71281 | 0.54552 | 0.55835 | 0.51347 | 0.54917 | 0.51282 |
| hex_Ti10AlV (THz) | 0.24389 | 0.61529 | 0.22854 | 0.28719 | 0.36761 | 0.21371 | 0.22668 | 0.21972 | 0.18272 |
| hex_Ti10AlV deltaF 0K (eV) | 0.00063000 | 0.0027700 | 0.00063000 | 0.0013000 | 0.0017900 | 0.00090000 | 0.00090000 | 0.0010900 | 0.00070000 |
| hex_Ti10AlV deltaF 2000K (eV) | 0.010220 | 0.053830 | 0.010490 | 0.021400 | 0.030690 | 0.012930 | 0.013820 | 0.016960 | 0.010960 |
| hcp_Ti6Al2 (THz) | 0.24862 | 0.33832 | 0.33421 | 0.27713 | 0.25852 | 0.28419 | 0.25864 | 0.27331 | 0.26232 |
| hcp_Ti6Al2 deltaF 0K (eV) | 0.00065000 | 0.00021000 | 0.00031000 | 0.00032000 | 0.00088000 | 0.0013400 | 0.0011400 | 0.0013800 | 0.0012400 |
| hcp_Ti6Al2 deltaF 2000K (eV) | 0.013430 | 9.0000e-05 | 0.0024600 | 0.0070400 | 0.015810 | 0.023180 | 0.020490 | 0.024710 | 0.023230 |
| hcp_Ti6V2 (THz) | 0.66300 | 0.58589 | 0.85638 | 0.73742 | 0.85167 | 0.71437 | 0.73050 | 0.56172 | 0.55572 |
| hcp_Ti7V (THz) | 0.54477 | 0.52276 | 0.47926 | 0.44095 | 0.45168 | 0.44108 | 0.43478 | 0.43269 | 0.42067 |
| bcc_Ti6Al2 (THz) | 0.42823 | 0.98798 | 0.45399 | 0.70213 | 0.51351 | 0.44558 | 0.48243 | 0.53283 | 0.50848 |
| bcc_Ti6V2 (THz) | 0.67420 | 1.1767 | 0.98953 | 0.79378 | 0.54173 | 0.67014 | 0.55000 | 0.49366 | 0.66819 |
| hex_Ti10Al2 (THz) | 0.31694 | 0.34013 | 0.29478 | 0.21488 | 0.19483 | 0.17574 | 0.17734 | 0.15868 | 0.18172 |
| hex_Ti10Al2 deltaF 0K (eV) | 0.00010000 | 0.00047000 | 0.00078000 | 1.0000e-05 | 0.00022000 | 0.00017000 | 0.00024000 | 0.00020000 | 0.00016000 |
| hex_Ti10Al2 deltaF 2000K (eV) | 0.056440 | 0.0031600 | 0.011160 | 0.00010000 | 0.0054700 | 0.0044100 | 0.0054800 | 0.0035900 | 0.0040700 |
| hex_Ti10V2 (THz) | 0.25425 | 0.34177 | 0.22861 | 0.19451 | 0.19768 | 0.18011 | 0.17198 | 0.17129 | 0.19272 |
| hex_Ti10V2 deltaF 0K (eV) | 0.00034000 | 0.00093000 | 0.00012000 | 0.00017000 | 0.00012000 | 2.0000e-05 | 2.0000e-05 | 0.00026000 | 0.00034000 |
| hex_Ti10V2 deltaF 2000K (eV) | 0.0088300 | 0.017880 | 0.0028100 | 0.0018000 | 0.0015500 | 0.0046300 | 0.0025200 | 0.0060500 | 0.0031100 |
| bcc_C11 (GPa) | 1.1800 | 10.890 | 6.9600 | 8.6200 | 2.7400 | 10.310 | 7.0900 | 3.2400 | 3.8600 |
| bcc_C12 (GPa) | 6.5600 | 5.0900 | 3.5600 | 6.3300 | 1.7500 | 6.0200 | 2.0400 | 0.58000 | 0.29000 |
| bcc_C44 (GPa) | 0.30000 | 8.6400 | 9.5800 | 7.3900 | 10.010 | 5.8100 | 5.9100 | 6.7600 | 5.5800 |
| bcc_B (GPa) | 3.9800 | 7.0233 | 4.6933 | 7.0933 | 2.0800 | 7.4500 | 3.7233 | 0.69333 | 1.0933 |
| bcc_EC_RMSE (GPa) | 3.8845 | 8.1926 | 6.6128 | 7.4044 | 5.3642 | 7.6124 | 5.0799 | 3.7753 | 3.4393 |
| hcp_C11 (GPa) | 1.1800 | 15.660 | 2.5800 | 4.3800 | 2.1700 | 0.69000 | 0.23000 | 6.5700 | 5.5400 |
| hcp_C12 (GPa) | 1.7700 | 4.2000 | 6.2300 | 8.7100 | 10.770 | 8.7600 | 9.8300 | 9.3200 | 10.830 |
| hcp_C13 (GPa) | 3.2900 | 13.570 | 10.240 | 13.910 | 9.4700 | 9.1100 | 8.3700 | 6.7300 | 6.5400 |
| hcp_C14 (GPa) | 0.66000 | 1.0600 | 0.54000 | 0.17000 | 0.38000 | 0.040000 | 0.030000 | 0.14000 | 0.080000 |
| hcp_C15 (GPa) | 1.1800 | 0.30000 | 0.060000 | 0.24000 | 0.57000 | 0.18000 | 0.34000 | 0.12000 | 0.020000 |
| hcp_C33 (GPa) | 10.190 | 1.0200 | 5.0900 | 8.1300 | 9.8300 | 4.9700 | 7.0100 | 2.8600 | 0.73000 |
| hcp_C44 (GPa) | 0.76000 | 13.320 | 7.3200 | 8.9300 | 12.350 | 8.6900 | 9.4300 | 10.830 | 10.380 |
| hcp_C66 (GPa) | 1.4800 | 9.9300 | 4.4000 | 6.5400 | 6.4700 | 4.0300 | 5.0300 | 7.9400 | 8.1800 |
| hcp_B (GPa) | 0.54466 | 8.4546 | 4.3654 | 6.3104 | 1.1811 | 1.3969 | 0.79210 | 2.0363 | 1.6356 |
| hcp_EC_RMSE (GPa) | 3.7130 | 9.4091 | 5.4623 | 7.5796 | 7.4778 | 5.5643 | 6.0631 | 6.3945 | 6.3999 |
| hex_C11 (GPa) | 5.2300 | 7.1600 | 4.4200 | 0.57000 | 4.1400 | 1.5500 | 3.3800 | 2.6000 | 0.040000 |
| hex_C12 (GPa) | 0.36000 | 5.6200 | 4.1200 | 4.2500 | 10.020 | 1.6500 | 3.1200 | 3.2900 | 3.2600 |
| hex_C13 (GPa) | 0.75000 | 0.070000 | 3.5700 | 8.0600 | 12.510 | 8.6400 | 2.3100 | 4.9400 | 9.5500 |
| hex_C14 (GPa) | 0.75000 | 0.60000 | 0.42000 | 0.75000 | 0.52000 | 0.25000 | 0.53000 | 0.53000 | 0.53000 |
| hex_C15 (GPa) | 0.21000 | 0.20000 | 0.28000 | 0.18000 | 0.22000 | 0.20000 | 0.020000 | 0.010000 | 0.37000 |
| hex_C33 (GPa) | 13.530 | 24.160 | 26.050 | 18.430 | 14.430 | 18.830 | 10.080 | 13.550 | 17.300 |
| hex_C44 (GPa) | 0.24000 | 17.270 | 10.220 | 8.1900 | 10.740 | 8.1400 | 8.4900 | 7.1300 | 10.100 |
| hex_C66 (GPa) | 2.4400 | 6.3900 | 0.15000 | 2.4100 | 2.9400 | 0.050000 | 3.2500 | 2.9400 | 1.6500 |
| hex_B (GPa) | 3.0684 | 1.9910 | 3.0726 | 0.76129 | 0.81102 | 1.0839 | 0.028330 | 0.59676 | 1.6323 |
| hex_EC_RMSE (GPa) | 5.0237 | 10.594 | 9.6726 | 7.4317 | 8.2140 | 7.4676 | 4.8422 | 5.6343 | 7.5199 |
| Mean Phonon RMSE (THz) | 0.42740 | 0.60728 | 0.47639 | 0.46232 | 0.42143 | 0.40482 | 0.38576 | 0.37068 | 0.37949 |
| Mean EC RMSE (GPa) | 4.2071 | 9.3984 | 7.2492 | 7.4719 | 7.0187 | 6.8814 | 5.3284 | 5.2681 | 5.7863 |
| Mean FOM | 0.71985 | 0.15768 | 0.51183 | 0.51453 | 0.66935 | 0.74979 | 0.79473 | 0.79149 | 0.81323 |
| FOM Tests Only | 0.65181 | 0.16614 | 0.51446 | 0.60796 | 0.64586 | 0.71429 | 0.76176 | 0.77057 | 0.77028 |

FIG. S6. **Freeze=5 Ti-Al-V models evaluated on the Ti-Al-V benchmarks**. The results are summarised in the figure of merit (FOM), which measure the performance of models on all benchmarks, relative to their alternatives. The models are trained on the different % of data, as outlined in the column titles. Details on the generation of this dataset can be found in Ref. [3].

| | ACE | MACE-MP-f 2% | MACE-MP-f 5% | MACE-MP-f 10% | MACE-MP-f 20% | MACE-MP-f 40% | MACE-MP-f 60% | MACE-MP-f 80% | MACE-MP-f 100% |
|---|---|---|---|---|---|---|---|---|---|
| E_train (eV) | 0.0037500 | 0.010720 | 0.0077300 | 0.0052100 | 0.0037200 | 0.0029000 | 0.0024700 | 0.0021600 | 0.0020100 |
| F_train (eV/AA) | 0.14928 | 0.28467 | 0.25937 | 0.22456 | 0.18997 | 0.14224 | 0.10710 | 0.086920 | 0.064080 |
| V_train (eV) | 0.024130 | 0.063160 | 0.056280 | 0.048630 | 0.041080 | 0.031970 | 0.030530 | 0.028700 | 0.027830 |
| E_validation (eV) | 0.0024700 | 0.0032200 | 0.0018600 | 0.0018000 | 0.0012700 | 0.0011300 | 0.0010100 | 0.00094000 | 0.00088000 |
| F_validation (eV/AA) | 0.12993 | 0.13650 | 0.11489 | 0.10391 | 0.091810 | 0.087770 | 0.085020 | 0.084650 | 0.081900 |
| V_validation (eV) | 0.011540 | 0.019860 | 0.012780 | 0.013590 | 0.011360 | 0.010060 | 0.010030 | 0.0095900 | 0.0086600 |
| hcp_Ti7AlV (THz) | 0.25810 | 0.48187 | 0.29881 | 0.23682 | 0.20558 | 0.23114 | 0.24664 | 0.30595 | 0.18264 |
| hcp_Ti7AlV deltaF 0K (eV) | 0.00038000 | 0.00069000 | 0.00022000 | 0.00026000 | 0.00047000 | 0.00068000 | 0.00021000 | 0.0011200 | 0.00080000 |
| hcp_Ti7AlV deltaF 2000K (eV) | 0.025320 | 0.029900 | 0.024650 | 0.015970 | 0.016670 | 0.012550 | 0.021720 | 0.030110 | 0.011110 |
| bcc_Ti7AlV (THz) | 0.64197 | 0.56763 | 0.59581 | 0.70464 | 0.54964 | 0.54334 | 0.56719 | 0.62080 | 0.60103 |
| hex_Ti10AlV (THz) | 0.24389 | 0.18544 | 0.12065 | 0.13711 | 0.25306 | 0.18092 | 0.17684 | 0.14969 | 0.14871 |
| hex_Ti10AlV deltaF 0K (eV) | 0.00063000 | 0.00066000 | 0.00011000 | 3.0000e-05 | 0.0011600 | 0.00081000 | 0.00054000 | 0.00045000 | 0.00048000 |
| hex_Ti10AlV deltaF 2000K (eV) | 0.010220 | 0.0095900 | 0.00054000 | 0.0011900 | 0.020060 | 0.011340 | 0.0093500 | 0.0065100 | 0.0085600 |
| hcp_Ti6Al2 (THz) | 0.24862 | 0.31508 | 0.36567 | 0.24518 | 0.18268 | 0.20688 | 0.24919 | 0.20130 | 0.20955 |
| hcp_Ti6Al2 deltaF 0K (eV) | 0.00065000 | 6.0000e-05 | 0.00054000 | 0.00044000 | 0.00061000 | 0.00018000 | 0.00017000 | 0.00067000 | 0.00016000 |
| hcp_Ti6Al2 deltaF 2000K (eV) | 0.013430 | 0.0059800 | 0.0020100 | 0.0085900 | 0.012540 | 0.0072600 | 0.0011600 | 0.011100 | 0.00019000 |
| hcp_Ti6V2 (THz) | 0.66300 | 0.57432 | 0.89898 | 0.97656 | 0.50030 | 0.45308 | 0.65183 | 0.44093 | 0.55269 |
| hcp_Ti7V (THz) | 0.54477 | 0.58814 | 0.51760 | 0.50972 | 0.54488 | 0.36389 | 0.42009 | 0.39962 | 0.41376 |
| bcc_Ti6Al2 (THz) | 0.42823 | 0.44942 | 0.48618 | 0.39325 | 0.40502 | 0.32210 | 0.42584 | 0.37711 | 0.31839 |
| bcc_Ti6V2 (THz) | 0.67420 | 0.70505 | 0.68270 | 0.51492 | 0.60661 | 0.81554 | 0.49462 | 0.51225 | 0.47840 |
| hex_Ti10Al2 (THz) | 0.31694 | 0.23379 | 0.41457 | 0.21444 | 0.62022 | 0.41902 | 0.16824 | 0.44849 | 0.48546 |
| hex_Ti10Al2 deltaF 0K (eV) | 0.00010000 | 0.00025000 | 0.0016300 | 0.00013000 | 0.0027900 | 0.0015800 | 0.00020000 | 0.0018400 | 0.0021600 |
| hex_Ti10Al2 deltaF 2000K (eV) | 0.056440 | 0.00086000 | 0.025120 | 0.069680 | 0.19537 | 0.023230 | 0.0045600 | 0.029760 | 0.035690 |
| hex_Ti10V2 (THz) | 0.25425 | 0.28055 | 0.78145 | 0.16889 | 0.16617 | 0.26250 | 0.14299 | 0.14574 | 0.14026 |
| hex_Ti10V2 deltaF 0K (eV) | 0.00034000 | 0.0012800 | 6.0000e-05 | 8.0000e-05 | 0.00015000 | 0.00049000 | 0.00020000 | 0.00064000 | 0.00010000 |
| hex_Ti10V2 deltaF 2000K (eV) | 0.0088300 | 0.020860 | 0.050890 | 0.00025000 | 0.0049200 | 0.012750 | 0.0027800 | 0.011920 | 0.0024200 |
| bcc_C11 (GPa) | 1.1800 | 5.0200 | 0.78000 | 5.7100 | 5.5500 | 3.4100 | 1.2200 | 4.4700 | 4.1200 |
| bcc_C12 (GPa) | 6.5600 | 1.6500 | 1.8500 | 2.0100 | 4.7700 | 0.60000 | 0.50000 | 0.23000 | 4.1000 |
| bcc_C44 (GPa) | 0.30000 | 8.7600 | 5.0200 | 5.6200 | 5.6200 | 6.8000 | 8.5200 | 6.6500 | 8.6800 |
| bcc_B (GPa) | 3.9800 | 0.57333 | 1.4933 | 0.56333 | 1.3300 | 0.73667 | 0.74000 | 1.6433 | 1.3600 |
| bcc_EC_RMSE (GPa) | 3.8845 | 5.1232 | 2.8045 | 4.1396 | 4.6612 | 3.8331 | 4.3266 | 4.0914 | 5.2673 |
| hcp_C11 (GPa) | 1.1800 | 8.6700 | 2.9000 | 0.45000 | 0.48000 | 0.69000 | 0.41000 | 0.40000 | 0.26000 |
| hcp_C12 (GPa) | 1.7700 | 10.780 | 9.3000 | 3.4200 | 8.8800 | 10.360 | 5.2500 | 3.4100 | 6.9000 |
| hcp_C13 (GPa) | 3.2900 | 5.2000 | 5.9700 | 4.6300 | 8.0000 | 5.9900 | 2.0300 | 0.65000 | 1.3800 |
| hcp_C14 (GPa) | 0.66000 | 0.72000 | 1.3200 | 0.42000 | 0.86000 | 0.050000 | 0.93000 | 0.36000 | 0.25000 |
| hcp_C15 (GPa) | 1.1800 | 1.4900 | 0.52000 | 0.83000 | 0.77000 | 0.26000 | 1.3400 | 0.56000 | 0.13000 |
| hcp_C33 (GPa) | 10.190 | 5.2800 | 28.830 | 16.680 | 16.510 | 8.6300 | 7.0800 | 10.860 | 4.3100 |
| hcp_C44 (GPa) | 0.76000 | 3.5800 | 1.6200 | 2.4900 | 0.14000 | 2.2000 | 0.85000 | 0.59000 | 1.1900 |
| hcp_C66 (GPa) | 1.4800 | 9.7200 | 3.2000 | 1.9300 | 4.2000 | 4.8300 | 2.8300 | 1.9000 | 3.5800 |
| hcp_B (GPa) | 0.54466 | 1.2409 | 3.1442 | 0.35283 | 0.36850 | 0.77629 | 2.7216 | 1.5061 | 1.3624 |
| hcp_EC_RMSE (GPa) | 3.7130 | 6.3058 | 10.469 | 5.9858 | 6.9506 | 5.2388 | 3.3462 | 3.8990 | 3.0610 |
| hex_C11 (GPa) | 5.2300 | 2.3800 | 3.1500 | 5.2800 | 3.5900 | 0.25000 | 2.8700 | 0.90000 | 0.54000 |
| hex_C12 (GPa) | 0.36000 | 2.5700 | 2.0800 | 2.4600 | 1.2400 | 2.4900 | 3.6600 | 0.16000 | 2.4200 |
| hex_C13 (GPa) | 0.75000 | 1.1500 | 0.010000 | 2.6200 | 0.93000 | 1.8000 | 0.45000 | 0.25000 | 0.29000 |
| hex_C14 (GPa) | 0.75000 | 0.090000 | 0.56000 | 0.22000 | 0.0000 | 0.98000 | 0.44000 | 0.65000 | 0.22000 |
| hex_C15 (GPa) | 0.21000 | 1.1200 | 0.050000 | 0.71000 | 0.60000 | 0.14000 | 0.12000 | 0.080000 | 0.43000 |
| hex_C33 (GPa) | 13.530 | 4.9200 | 1.2600 | 10.470 | 6.1200 | 2.6000 | 7.1200 | 2.9300 | 0.89000 |
| hex_C44 (GPa) | 0.24000 | 1.6000 | 2.1400 | 1.8400 | 0.40000 | 0.18000 | 0.61000 | 1.6600 | 0.44000 |
| hex_C66 (GPa) | 2.4400 | 2.4800 | 2.6100 | 1.4100 | 1.1700 | 1.1200 | 0.40000 | 0.53000 | 0.94000 |
| hex_B (GPa) | 3.0684 | 0.032240 | 0.11150 | 1.7194 | 1.3388 | 0.10605 | 2.4379 | 0.37089 | 0.64238 |
| hex_EC_RMSE (GPa) | 5.0237 | 2.3039 | 1.7498 | 4.2071 | 2.5039 | 1.4353 | 2.9667 | 1.2058 | 0.98586 |
| Mean Phonon RMSE (THz) | 0.42740 | 0.43813 | 0.51624 | 0.41015 | 0.40342 | 0.37984 | 0.35435 | 0.36019 | 0.35309 |
| Mean EC RMSE (GPa) | 4.2071 | 4.5776 | 5.0079 | 4.7775 | 4.7052 | 3.5024 | 3.5465 | 3.0654 | 3.1047 |
| Mean FOM | 0.57032 | 0.22651 | 0.44329 | 0.55579 | 0.61177 | 0.75241 | 0.79579 | 0.79963 | 0.86504 |
| FOM Tests Only | 0.49215 | 0.30201 | 0.52052 | 0.59908 | 0.61580 | 0.74291 | 0.77345 | 0.75924 | 0.83058 |

FIG. S7. **Freeze=4 Ti-Al-V models evaluated on the Ti-Al-V benchmarks**. The results are summarised in the figure of merit (FOM), which measure the performance of models on all benchmarks, relative to their alternatives. The models are trained on the different % of data, as outlined in the column titles. Details on the generation of this dataset can be found in Ref. [3].

| | ACE | MACE 10-1 | MACE 10-2 | MACE 10-3 | MACE 10-4 | MACE 10-5 | MACE 10 ensemble |
|---|---|---|---|---|---|---|---|
| E_train (eV) | 0.0037500 | 0.0052900 | 0.0051400 | 0.0049100 | 0.0052100 | 0.0050200 | 0.0051200 |
| F_train (eV/AA) | 0.14928 | 0.23225 | 0.19523 | 0.21216 | 0.22456 | 0.21977 | 0.21679 |
| V_train (eV) | 0.024130 | 0.050500 | 0.050960 | 0.049380 | 0.048630 | 0.049650 | 0.049820 |
| E_validation (eV) | 0.0024700 | 0.0051900 | 0.0020200 | 0.0023100 | 0.0018000 | 0.0016700 | 0.0026000 |
| F_validation (eV/AA) | 0.12993 | 0.20524 | 0.10487 | 0.10433 | 0.10391 | 0.10475 | 0.12462 |
| V_validation (eV) | 0.011540 | 0.050670 | 0.012740 | 0.014390 | 0.013590 | 0.012720 | 0.020820 |
| hcp_Ti7AlV (THz) | 0.25810 | 0.19252 | 0.23407 | 0.19901 | 0.23682 | 0.21956 | 0.21640 |
| hcp_Ti7AlV deltaF 0K (eV) | 0.00038000 | 0.00064000 | 0.00014000 | 0.00017000 | 0.00026000 | 8.0000e-05 | 0.00026000 |
| hcp_Ti7AlV deltaF 2000K (eV) | 0.025320 | 0.011820 | 0.022880 | 0.017910 | 0.015970 | 0.019740 | 0.017660 |
| bcc_Ti7AlV (THz) | 0.64197 | 0.55503 | 0.64974 | 0.61576 | 0.70464 | 0.58708 | 0.62245 |
| hex_Ti10AlV (THz) | 0.24389 | 0.17116 | 0.21369 | 0.25633 | 0.13711 | 0.16621 | 0.18890 |
| hex_Ti10AlV deltaF 0K (eV) | 0.00063000 | 0.00055000 | 0.00090000 | 0.0011900 | 3.0000e-05 | 0.00027000 | 0.00059000 |
| hex_Ti10AlV deltaF 2000K (eV) | 0.010220 | 0.010470 | 0.014610 | 0.022170 | 0.0011900 | 0.0057000 | 0.010830 |
| hcp_Ti6Al2 (THz) | 0.24862 | 0.22698 | 0.23677 | 0.21022 | 0.24518 | 0.28144 | 0.24012 |
| hcp_Ti6Al2 deltaF 0K (eV) | 0.00065000 | 0.00042000 | 0.00024000 | 0.00041000 | 0.00044000 | 0.00026000 | 0.00036000 |
| hcp_Ti6Al2 deltaF 2000K (eV) | 0.013430 | 0.0019900 | 0.00082000 | 0.0098700 | 0.0085900 | 0.0020800 | 0.0046700 |
| hcp_Ti6V2 (THz) | 0.66300 | 0.76274 | 0.47452 | 0.56283 | 0.97656 | 0.64296 | 0.68392 |
| hcp_Ti7V (THz) | 0.54477 | 0.49788 | 0.50233 | 0.45104 | 0.50972 | 0.45797 | 0.48379 |
| bcc_Ti6Al2 (THz) | 0.42823 | 0.33561 | 0.43221 | 0.36338 | 0.39325 | 0.44832 | 0.39455 |
| bcc_Ti6V2 (THz) | 0.67420 | 0.76279 | 0.48648 | 0.56680 | 0.51492 | 0.62842 | 0.59188 |
| hex_Ti10Al2 (THz) | 0.31694 | 0.44937 | 0.20193 | 0.47307 | 0.21444 | 0.45442 | 0.35864 |
| hex_Ti10Al2 deltaF 0K (eV) | 0.00010000 | 0.0020600 | 0.00059000 | 0.0021300 | 0.00013000 | 0.0019100 | 0.0013600 |
| hex_Ti10Al2 deltaF 2000K (eV) | 0.056440 | 0.030880 | 0.0072500 | 0.030910 | 0.069680 | 0.029240 | 0.033590 |
| hex_Ti10V2 (THz) | 0.25425 | 0.20547 | 0.16523 | 0.17273 | 0.16889 | 0.28085 | 0.19863 |
| hex_Ti10V2 deltaF 0K (eV) | 0.00034000 | 0.00028000 | 7.0000e-05 | 0.00016000 | 8.0000e-05 | 0.00040000 | 0.00020000 |
| hex_Ti10V2 deltaF 2000K (eV) | 0.0088300 | 0.0061900 | 0.0016900 | 0.0036400 | 0.00025000 | 0.013490 | 0.0050500 |
| bcc_C11 (GPa) | 1.1800 | 1.8600 | 5.0400 | 0.84000 | 5.7100 | 1.5200 | 2.9940 |
| bcc_C12 (GPa) | 6.5600 | 1.2700 | 0.50000 | 4.2000 | 2.0100 | 1.2600 | 1.8480 |
| bcc_C44 (GPa) | 0.30000 | 5.0400 | 11.030 | 4.8700 | 5.6200 | 5.2200 | 6.3560 |
| bcc_B (GPa) | 3.9800 | 0.22667 | 1.3467 | 2.5200 | 0.56333 | 1.3467 | 1.2007 |
| bcc_EC_RMSE (GPa) | 3.8845 | 2.7625 | 6.1059 | 3.4790 | 4.1396 | 2.8705 | 3.8715 |
| hcp_C11 (GPa) | 1.1800 | 1.7600 | 1.6300 | 0.17000 | 0.45000 | 1.4600 | 1.0940 |
| hcp_C12 (GPa) | 1.7700 | 6.5100 | 4.6000 | 4.0900 | 3.4200 | 5.4100 | 4.8060 |
| hcp_C13 (GPa) | 3.2900 | 0.19000 | 3.3800 | 1.4700 | 4.6300 | 6.2400 | 3.1820 |
| hcp_C14 (GPa) | 0.66000 | 0.31000 | 1.5600 | 0.23000 | 0.42000 | 0.78000 | 0.66000 |
| hcp_C15 (GPa) | 1.1800 | 1.4800 | 1.1000 | 1.1100 | 0.83000 | 0.90000 | 1.0840 |
| hcp_C33 (GPa) | 10.190 | 13.380 | 11.720 | 16.960 | 16.680 | 13.400 | 14.428 |
| hcp_C44 (GPa) | 0.76000 | 0.72000 | 0.050000 | 1.5600 | 2.4900 | 0.54000 | 1.0720 |
| hcp_C66 (GPa) | 1.4800 | 2.3800 | 3.1100 | 2.1300 | 1.9300 | 3.4400 | 2.5980 |
| hcp_B (GPa) | 0.54466 | 3.1934 | 0.40839 | 1.9505 | 0.35283 | 0.43076 | 1.2672 |
| hcp_EC_RMSE (GPa) | 3.7130 | 5.1983 | 4.5471 | 5.9500 | 5.9858 | 5.4122 | 5.4187 |
| hex_C11 (GPa) | 5.2300 | 0.19000 | 1.3600 | 1.9800 | 5.2800 | 1.1900 | 2.0000 |
| hex_C12 (GPa) | 0.36000 | 0.25000 | 3.2000 | 0.61000 | 2.4600 | 3.8000 | 2.0640 |
| hex_C13 (GPa) | 0.75000 | 2.1700 | 2.6600 | 1.4700 | 2.6200 | 2.0700 | 2.1980 |
| hex_C14 (GPa) | 0.75000 | 0.55000 | 0.64000 | 0.42000 | 0.22000 | 0.46000 | 0.45800 |
| hex_C15 (GPa) | 0.21000 | 0.42000 | 0.25000 | 0.70000 | 0.71000 | 0.21000 | 0.45800 |
| hex_C33 (GPa) | 13.530 | 1.1400 | 9.7800 | 3.1500 | 10.470 | 1.1800 | 5.1440 |
| hex_C44 (GPa) | 0.24000 | 1.2400 | 0.84000 | 0.30000 | 1.8400 | 2.2100 | 1.2860 |
| hex_C66 (GPa) | 2.4400 | 0.22000 | 2.2800 | 0.69000 | 1.4100 | 1.3000 | 1.1800 |
| hex_B (GPa) | 3.0684 | 1.0831 | 1.7020 | 0.36866 | 1.7194 | 0.069600 | 0.98855 |
| hex_EC_RMSE (GPa) | 5.0237 | 1.0190 | 3.7131 | 1.4041 | 4.2071 | 1.7754 | 2.4238 |
| Mean Phonon RMSE (THz) | 0.42740 | 0.41595 | 0.35970 | 0.38712 | 0.41015 | 0.41672 | 0.39793 |
| Mean EC RMSE (GPa) | 4.2071 | 2.9933 | 4.7887 | 3.6110 | 4.7775 | 3.3527 | 3.9047 |
| FOM | 0.49519 | 0.53136 | 0.54566 | 0.59145 | 0.51462 | 0.53881 | 0.54438 |

FIG. S8. **Freeze=4 10% ensemble of models compared with ACE-C, using an ensemble average**. This figure quantified uncertainty of the models by training on the randomly uniformly sampled 10% subsets of the full training set. Details on the generation of this dataset can be found in Ref. [3].

| | ACE | MACE 2% | MACE-MP-f %2 | MACE 5% | MACE-MP-f 5% | MACE 10% | MACE-MP-f 10% | MACE 20% |
|---|---|---|---|---|---|---|---|---|
| E_train (eV) | 0.0037500 | 0.011010 | 0.010720 | 0.0076500 | 0.0077300 | 0.0043100 | 0.0052100 | 0.0062500 |
| F_train (eV/AA) | 0.14928 | 0.29011 | 0.28467 | 0.26280 | 0.25937 | 0.19661 | 0.22456 | 0.25126 |
| V_train (eV) | 0.024130 | 0.066160 | 0.063160 | 0.053520 | 0.056280 | 0.041720 | 0.048630 | 0.045830 |
| E_validation (eV) | 0.0024700 | 0.0029900 | 0.0032200 | 0.0017800 | 0.0018600 | 0.0015000 | 0.0018000 | 0.0010700 |
| F_validation (eV/AA) | 0.12993 | 0.13979 | 0.13650 | 0.11961 | 0.11489 | 0.10593 | 0.10391 | 0.097990 |
| V_validation (eV) | 0.011540 | 0.022840 | 0.019860 | 0.014090 | 0.012780 | 0.011710 | 0.013590 | 0.012020 |
| hcp_Ti7AlV (THz) | 0.25810 | 0.36985 | 0.48187 | 0.30756 | 0.29881 | 0.27902 | 0.23682 | 0.36287 |
| hcp_Ti7AlV deltaF 0K (eV) | 0.00038000 | 0.0013100 | 0.00069000 | 0.00082000 | 0.00022000 | 0.00048000 | 0.00026000 | 0.00041000 |
| hcp_Ti7AlV deltaF 2000K (eV) | 0.025320 | 0.046390 | 0.029900 | 0.0063300 | 0.024650 | 0.021680 | 0.015970 | 0.025150 |
| bcc_Ti7AlV (THz) | 0.64197 | 0.66254 | 0.56763 | 0.66608 | 0.59581 | 0.50720 | 0.70464 | 0.64434 |
| hex_Ti10AlV (THz) | 0.24389 | 0.21930 | 0.18544 | 0.19711 | 0.12065 | 0.16048 | 0.13711 | 0.28162 |
| hex_Ti10AlV deltaF 0K (eV) | 0.00063000 | 0.00068000 | 0.00066000 | 0.00069000 | 0.00011000 | 0.00058000 | 3.0000e-05 | 0.0014200 |
| hex_Ti10AlV deltaF 2000K (eV) | 0.010220 | 0.013530 | 0.0095900 | 0.014070 | 0.00054000 | 0.0099800 | 0.0011900 | 0.025550 |
| hcp_Ti6Al2 (THz) | 0.24862 | 0.37014 | 0.31508 | 0.29260 | 0.36567 | 0.22787 | 0.24518 | 0.23987 |
| hcp_Ti6Al2 deltaF 0K (eV) | 0.00065000 | 0.0014500 | 6.0000e-05 | 0.00057000 | 0.00054000 | 0.00064000 | 0.00044000 | 0.0011500 |
| hcp_Ti6Al2 deltaF 2000K (eV) | 0.013430 | 0.032740 | 0.0059800 | 0.0025100 | 0.0020100 | 0.015150 | 0.0085900 | 0.023880 |
| hcp_Ti6V2 (THz) | 0.66300 | 0.61220 | 0.57432 | 0.58734 | 0.89898 | 1.1199 | 0.97656 | 0.67625 |
| hcp_Ti7V (THz) | 0.54477 | 0.51615 | 0.58814 | 0.47209 | 0.51760 | 0.44126 | 0.50972 | 0.46391 |
| bcc_Ti6Al2 (THz) | 0.42823 | 0.47901 | 0.44942 | 0.41916 | 0.48618 | 0.34259 | 0.39325 | 0.36294 |
| bcc_Ti6V2 (THz) | 0.67420 | 0.62118 | 0.70505 | 0.61232 | 0.68270 | 0.68720 | 0.51492 | 0.65829 |
| hex_Ti10Al2 (THz) | 0.31694 | 0.37589 | 0.23379 | 0.45277 | 0.41457 | 0.41620 | 0.21444 | 0.54792 |
| hex_Ti10Al2 deltaF 0K (eV) | 0.00010000 | 0.00047000 | 0.00025000 | 0.0017100 | 0.0016300 | 0.0015000 | 0.00013000 | 0.0026000 |
| hex_Ti10Al2 deltaF 2000K (eV) | 0.056440 | 0.041890 | 0.00086000 | 0.022360 | 0.025120 | 0.024690 | 0.069680 | 0.039760 |
| hex_Ti10V2 (THz) | 0.25425 | 0.23172 | 0.28055 | 0.53166 | 0.78145 | 0.18303 | 0.16889 | 0.18134 |
| hex_Ti10V2 deltaF 0K (eV) | 0.00034000 | 0.00028000 | 0.0012800 | 0.00098000 | 6.0000e-05 | 2.0000e-05 | 8.0000e-05 | 0.00059000 |
| hex_Ti10V2 deltaF 2000K (eV) | 0.0088300 | 0.00011000 | 0.020860 | 0.0015100 | 0.050890 | 0.0017300 | 0.00025000 | 0.010590 |
| bcc_C11 (GPa) | 1.1800 | 2.1300 | 5.0200 | 4.3000 | 0.78000 | 8.2700 | 5.7100 | 3.5700 |
| bcc_C12 (GPa) | 6.5600 | 0.52000 | 1.6500 | 0.31000 | 1.8500 | 2.7600 | 2.0100 | 6.9800 |
| bcc_C44 (GPa) | 0.30000 | 2.8200 | 8.7600 | 7.2200 | 5.0200 | 2.5100 | 5.6200 | 3.4000 |
| bcc_B (GPa) | 3.9800 | 1.0567 | 0.57333 | 1.6400 | 1.4933 | 4.5967 | 0.56333 | 5.8433 |
| bcc_EC_RMSE (GPa) | 3.8845 | 1.8625 | 5.1232 | 4.2838 | 2.8045 | 5.0853 | 4.1396 | 5.1761 |
| hcp_C11 (GPa) | 1.1800 | 15.930 | 8.6700 | 0.88000 | 2.9000 | 4.9300 | 0.45000 | 3.0700 |
| hcp_C12 (GPa) | 1.7700 | 0.090000 | 10.780 | 5.5700 | 9.3000 | 2.5700 | 3.4200 | 0.96000 |
| hcp_C13 (GPa) | 3.2900 | 6.7700 | 5.2000 | 1.6400 | 5.9700 | 1.9300 | 4.6300 | 5.9900 |
| hcp_C14 (GPa) | 0.66000 | 0.98000 | 0.72000 | 1.7000 | 1.3200 | 0.17000 | 0.42000 | 0.30000 |
| hcp_C15 (GPa) | 1.1800 | 1.1200 | 1.4900 | 0.97000 | 0.52000 | 0.16000 | 0.83000 | 0.63000 |
| hcp_C33 (GPa) | 10.190 | 19.510 | 5.2800 | 13.960 | 28.830 | 3.9200 | 16.680 | 9.5800 |
| hcp_C44 (GPa) | 0.76000 | 3.4700 | 3.5800 | 2.0500 | 1.6200 | 0.21000 | 2.4900 | 1.6600 |
| hcp_C66 (GPa) | 1.4800 | 7.9200 | 9.7200 | 2.3400 | 3.2000 | 1.1800 | 1.9300 | 2.0100 |
| hcp_B (GPa) | 0.54466 | 2.7683 | 1.2409 | 2.2299 | 3.1442 | 2.2154 | 0.35283 | 2.1224 |
| hcp_EC_RMSE (GPa) | 3.7130 | 9.2189 | 6.3058 | 5.2478 | 10.469 | 2.5033 | 5.9858 | 4.0796 |
| hex_C11 (GPa) | 5.2300 | 11.310 | 2.3800 | 12.460 | 3.1500 | 2.8800 | 5.2800 | 1.0200 |
| hex_C12 (GPa) | 0.36000 | 1.4100 | 2.5700 | 2.9500 | 2.0800 | 8.0200 | 2.4600 | 0.70000 |
| hex_C13 (GPa) | 0.75000 | 1.4200 | 1.1500 | 0.57000 | 0.010000 | 4.7600 | 2.6200 | 0.24000 |
| hex_C14 (GPa) | 0.75000 | 0.35000 | 0.090000 | 1.1600 | 0.56000 | 0.14000 | 0.22000 | 0.030000 |
| hex_C15 (GPa) | 0.21000 | 1.4100 | 1.1200 | 0.54000 | 0.050000 | 0.39000 | 0.71000 | 0.39000 |
| hex_C33 (GPa) | 13.530 | 0.32000 | 4.9200 | 4.1200 | 1.2600 | 8.8000 | 10.470 | 14.320 |
| hex_C44 (GPa) | 0.24000 | 3.9600 | 1.6000 | 5.0300 | 2.1400 | 0.68000 | 1.8400 | 1.0100 |
| hex_C66 (GPa) | 2.4400 | 6.3600 | 2.4800 | 4.7600 | 2.6100 | 2.5700 | 1.4100 | 0.86000 |
| hex_B (GPa) | 3.0684 | 1.7163 | 0.032240 | 3.7569 | 0.11150 | 0.80533 | 1.7194 | 1.7876 |
| hex_EC_RMSE (GPa) | 5.0237 | 4.6333 | 2.3039 | 5.2171 | 1.7498 | 4.4796 | 4.2071 | 4.8506 |
| Mean Phonon RMSE (THz) | 0.42740 | 0.44580 | 0.43813 | 0.45387 | 0.51624 | 0.43648 | 0.41015 | 0.44194 |
| Mean EC RMSE (GPa) | 4.2071 | 5.2383 | 4.5776 | 4.9162 | 5.0079 | 4.0227 | 4.7775 | 4.7021 |
| Mean FOM | 0.61439 | 0.22856 | 0.30253 | 0.45195 | 0.49168 | 0.63964 | 0.60529 | 0.56216 |
| FOM Tests Only | 0.56089 | 0.30474 | 0.38972 | 0.51807 | 0.57769 | 0.66875 | 0.66418 | 0.62328 |

| | MACE-MP-f 20% | MACE 40% | MACE-MP-f 40% | MACE 60% | MACE-MP-f 60% | MACE 80% | MACE-MP-f 80% | MACE 100% | MACE-MP-f 100% |
|---|---|---|---|---|---|---|---|---|---|
| E_train (eV) | 0.0037200 | 0.0025400 | 0.0029000 | 0.0020000 | 0.0024700 | 0.0016400 | 0.0021600 | 0.0014200 | 0.0020100 |
| F_train (eV/AA) | 0.18997 | 0.14343 | 0.14224 | 0.097240 | 0.10710 | 0.091220 | 0.086920 | 0.041960 | 0.064080 |
| V_train (eV) | 0.041080 | 0.030160 | 0.031970 | 0.027470 | 0.030530 | 0.025180 | 0.028700 | 0.024720 | 0.027830 |
| E_validation (eV) | 0.0012700 | 0.00091000 | 0.0011300 | 0.00096000 | 0.0010100 | 0.00078000 | 0.00094000 | 0.00080000 | 0.00088000 |
| F_validation (eV/AA) | 0.091810 | 0.088710 | 0.087770 | 0.086450 | 0.085020 | 0.082590 | 0.084650 | 0.082550 | 0.081900 |
| V_validation (eV) | 0.011360 | 0.010230 | 0.010060 | 0.0092900 | 0.010030 | 0.0095900 | 0.0095900 | 0.0087500 | 0.0086600 |
| hcp_Ti7AlV (THz) | 0.20558 | 0.23820 | 0.23114 | 0.21558 | 0.24664 | 0.33567 | 0.30595 | 0.17960 | 0.18264 |
| hcp_Ti7AlV deltaF 0K (eV) | 0.00047000 | 0.00072000 | 0.00068000 | 0.00098000 | 0.00021000 | 0.00034000 | 0.0011200 | 0.00072000 | 0.00080000 |
| hcp_Ti7AlV deltaF 2000K (eV) | 0.016670 | 0.0095700 | 0.012550 | 0.0065700 | 0.021720 | 0.013500 | 0.030110 | 0.0064500 | 0.011110 |
| bcc_Ti7AlV (THz) | 0.54964 | 0.54416 | 0.54334 | 0.54526 | 0.56719 | 0.60683 | 0.62080 | 0.57043 | 0.60103 |
| hex_Ti10AlV (THz) | 0.25306 | 0.15974 | 0.18092 | 0.15207 | 0.17684 | 0.14488 | 0.14969 | 0.12657 | 0.14871 |
| hex_Ti10AlV deltaF 0K (eV) | 0.0011600 | 0.00068000 | 0.00081000 | 0.00051000 | 0.00054000 | 0.00049000 | 0.00045000 | 0.00030000 | 0.00048000 |
| hex_Ti10AlV deltaF 2000K (eV) | 0.020060 | 0.012970 | 0.011340 | 0.0095600 | 0.0093500 | 0.0092900 | 0.0065100 | 0.0054500 | 0.0085600 |
| hcp_Ti6Al2 (THz) | 0.18268 | 0.21280 | 0.20688 | 0.21614 | 0.24919 | 0.26029 | 0.20130 | 0.23243 | 0.20955 |
| hcp_Ti6Al2 deltaF 0K (eV) | 0.00061000 | 4.0000e-05 | 0.00018000 | 0.00013000 | 0.00017000 | 0.00055000 | 0.00067000 | 0.00043000 | 0.00016000 |
| hcp_Ti6Al2 deltaF 2000K (eV) | 0.012540 | 0.0029400 | 0.0072600 | 0.00096000 | 0.0011600 | 0.0073500 | 0.011100 | 0.0053100 | 0.00019000 |
| hcp_Ti6V2 (THz) | 0.50030 | 0.55295 | 0.45308 | 0.85469 | 0.65183 | 0.55963 | 0.44093 | 0.60627 | 0.55269 |
| hcp_Ti7V (THz) | 0.54488 | 0.38437 | 0.36389 | 0.42118 | 0.42009 | 0.39640 | 0.39962 | 0.41108 | 0.41376 |
| bcc_Ti6Al2 (THz) | 0.40502 | 0.38639 | 0.32210 | 0.39433 | 0.42584 | 0.38053 | 0.37711 | 0.43861 | 0.31839 |
| bcc_Ti6V2 (THz) | 0.60661 | 0.67143 | 0.81554 | 0.70389 | 0.49462 | 0.75306 | 0.51225 | 0.47685 | 0.47840 |
| hex_Ti10Al2 (THz) | 0.62022 | 0.54379 | 0.41902 | 0.54205 | 0.16824 | 0.50404 | 0.44849 | 0.34915 | 0.48546 |
| hex_Ti10Al2 deltaF 0K (eV) | 0.0027900 | 0.0025700 | 0.0015800 | 0.0022900 | 0.00020000 | 0.0023300 | 0.0018400 | 0.0016000 | 0.0021600 |
| hex_Ti10Al2 deltaF 2000K (eV) | 0.19537 | 0.037730 | 0.023230 | 0.038220 | 0.0045600 | 0.035420 | 0.029760 | 0.15996 | 0.035690 |
| hex_Ti10V2 (THz) | 0.16617 | 0.18131 | 0.26250 | 0.17862 | 0.14299 | 0.16660 | 0.14574 | 0.13318 | 0.14026 |
| hex_Ti10V2 deltaF 0K (eV) | 0.00015000 | 7.0000e-05 | 0.00049000 | 0.00030000 | 0.00020000 | 0.00018000 | 0.00064000 | 2.0000e-05 | 0.00010000 |
| hex_Ti10V2 deltaF 2000K (eV) | 0.0049200 | 0.00032000 | 0.012750 | 0.0057700 | 0.0027800 | 0.0054000 | 0.011920 | 0.0035700 | 0.0024200 |
| bcc_C11 (GPa) | 5.5500 | 1.1900 | 3.4100 | 2.0700 | 1.2200 | 1.9500 | 4.4700 | 1.6500 | 4.1200 |
| bcc_C12 (GPa) | 4.7700 | 3.4900 | 0.60000 | 0.30000 | 0.50000 | 0.69000 | 0.23000 | 2.7100 | 4.1000 |
| bcc_C44 (GPa) | 5.6200 | 7.8300 | 6.8000 | 5.7200 | 8.5200 | 6.4400 | 6.6500 | 4.8800 | 8.6800 |
| bcc_B (GPa) | 1.3300 | 1.9300 | 0.73667 | 0.49000 | 0.74000 | 1.1100 | 1.6433 | 2.3567 | 1.3600 |
| bcc_EC_RMSE (GPa) | 4.6612 | 4.4337 | 3.8331 | 3.0551 | 4.3266 | 3.4272 | 4.0914 | 3.1399 | 5.2673 |
| hcp_C11 (GPa) | 0.48000 | 0.30000 | 0.69000 | 1.3300 | 0.41000 | 0.29000 | 0.40000 | 1.9100 | 0.26000 |
| hcp_C12 (GPa) | 8.8800 | 2.5000 | 10.360 | 2.1600 | 5.2500 | 5.1700 | 3.4100 | 0.79000 | 6.9000 |
| hcp_C13 (GPa) | 8.0000 | 0.15000 | 5.9900 | 2.5600 | 2.0300 | 0.60000 | 0.65000 | 0.83000 | 1.3800 |
| hcp_C14 (GPa) | 0.86000 | 0.41000 | 0.050000 | 0.43000 | 0.93000 | 0.59000 | 0.36000 | 0.73000 | 0.25000 |
| hcp_C15 (GPa) | 0.77000 | 0.34000 | 0.26000 | 0.70000 | 1.3400 | 1.0900 | 0.56000 | 0.88000 | 0.13000 |
| hcp_C33 (GPa) | 16.510 | 7.1600 | 8.6300 | 8.2300 | 7.0800 | 6.6000 | 10.860 | 1.4500 | 4.3100 |
| hcp_C44 (GPa) | 0.14000 | 1.2100 | 2.2000 | 0.68000 | 0.85000 | 0.16000 | 0.59000 | 1.8500 | 1.1900 |
| hcp_C66 (GPa) | 4.2000 | 1.4000 | 4.8300 | 0.41000 | 2.8300 | 2.7300 | 1.9000 | 1.3500 | 3.5800 |
| hcp_B (GPa) | 0.36850 | 1.2982 | 0.77629 | 2.7406 | 2.7216 | 1.5433 | 1.5061 | 0.77054 | 1.3624 |
| hcp_EC_RMSE (GPa) | 6.9506 | 2.6462 | 5.2388 | 3.1541 | 3.3462 | 3.0208 | 3.8990 | 1.2565 | 3.0610 |
| hex_C11 (GPa) | 3.5900 | 1.1300 | 0.25000 | 4.0500 | 2.8700 | 3.1300 | 0.90000 | 3.8800 | 0.54000 |
| hex_C12 (GPa) | 1.2400 | 1.3400 | 2.4900 | 1.3800 | 3.6600 | 2.7800 | 0.16000 | 1.6400 | 2.4200 |
| hex_C13 (GPa) | 0.93000 | 5.3400 | 1.8000 | 1.5800 | 0.45000 | 0.84000 | 0.25000 | 1.9700 | 0.29000 |
| hex_C14 (GPa) | 0.0000 | 0.030000 | 0.98000 | 0.020000 | 0.44000 | 0.71000 | 0.65000 | 0.37000 | 0.22000 |
| hex_C15 (GPa) | 0.60000 | 0.10000 | 0.14000 | 0.31000 | 0.12000 | 1.1200 | 0.080000 | 0.18000 | 0.43000 |
| hex_C33 (GPa) | 6.1200 | 2.4600 | 2.6000 | 3.9500 | 7.1200 | 11.040 | 2.9300 | 0.92000 | 0.89000 |
| hex_C44 (GPa) | 0.40000 | 0.25000 | 0.18000 | 0.070000 | 0.61000 | 0.42000 | 1.6600 | 1.8200 | 0.44000 |
| hex_C66 (GPa) | 1.1700 | 1.2400 | 1.1200 | 1.3400 | 0.40000 | 0.17000 | 0.53000 | 1.1200 | 0.94000 |
| hex_B (GPa) | 1.3388 | 2.1362 | 0.10605 | 2.3475 | 2.4379 | 2.1397 | 0.37089 | 2.2206 | 0.64238 |
| hex_EC_RMSE (GPa) | 2.5039 | 2.2064 | 1.4353 | 2.2064 | 2.9667 | 4.0367 | 1.2058 | 1.8897 | 0.98586 |
| Mean Phonon RMSE (THz) | 0.40342 | 0.38752 | 0.37984 | 0.42238 | 0.35435 | 0.41079 | 0.36019 | 0.35242 | 0.35309 |
| Mean EC RMSE (GPa) | 4.7052 | 3.0954 | 3.5024 | 2.8052 | 3.5465 | 3.4949 | 3.0654 | 2.0954 | 3.1047 |
| Mean FOM | 0.64772 | 0.78749 | 0.76330 | 0.81259 | 0.80389 | 0.80962 | 0.81584 | 0.86958 | 0.86031 |
| FOM Tests Only | 0.66803 | 0.79100 | 0.76719 | 0.79040 | 0.79679 | 0.77345 | 0.79524 | 0.82767 | 0.84029 |

FIG. S9. **Freeze=4 comparred with MACE from-scratch Ti-Al-V models evaluated on the Ti-Al-V benchmarks**. The results are summarised in the figure of merit (FOM), which measure the performance of models on all benchmarks, relative to their alternatives. The models are trained on the different % of data, as outlined in the column titles. Details on the generation of this dataset can be found in Ref. [3].

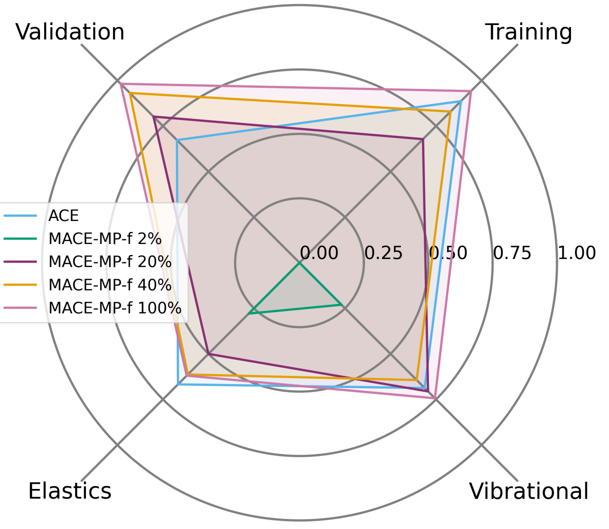


FIG. S10. **Figures of merit (FOM) for freeze=5 models relative to the custom ACE**. This figure summarises the comparison of predictive performance across a list of benchmarks in Fig.S6.

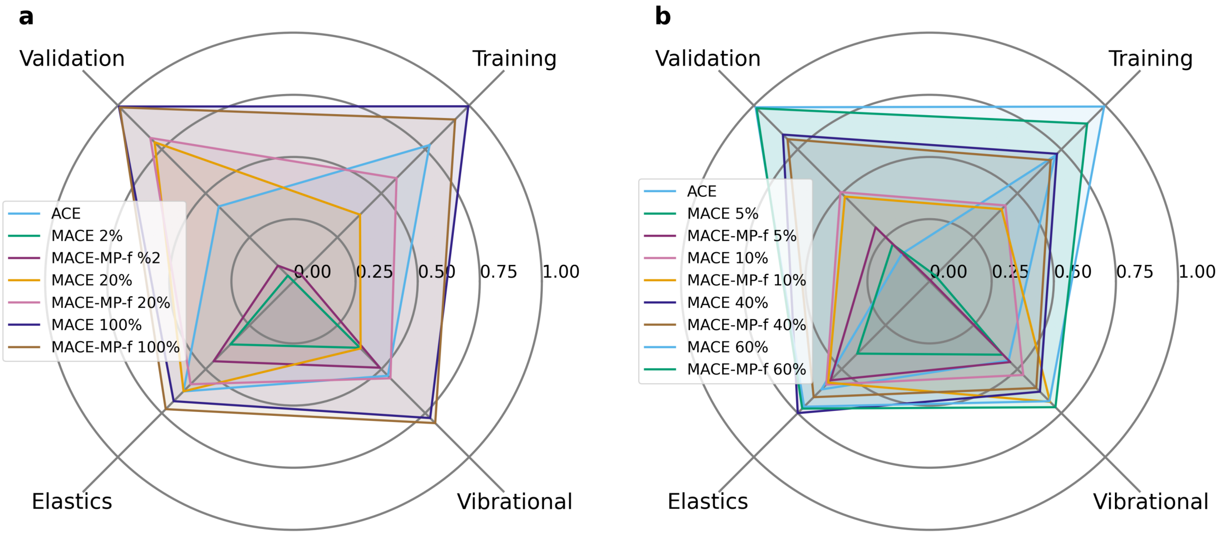


FIG. S11. **Figures of merit (FOM) for freeze=4 models against the from-scratch MACE models.**: Panel (a) shows the training subsets of 2, 20 and 100%. Panel (b) shows training subsets of 5, 10, 40 and 60%. These figures summarise the comparison of predictive performance across a list of benchmarks in Fig.S9.

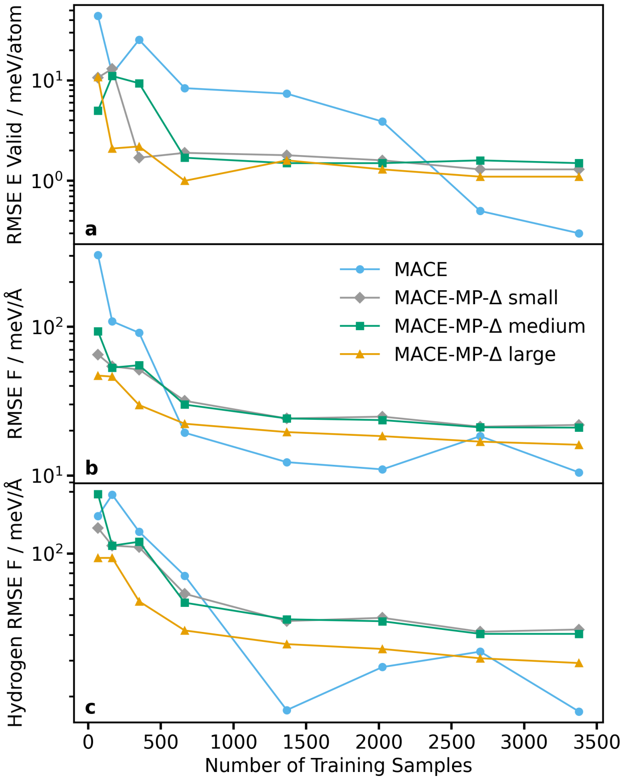


FIG. S12. **Delta learning curves for Hydrogen on Copper surface**. These delta models were trained based on MACE-MP "small" (grey diamonds), "medium" (green squares), and "large" (yellow triangles) foundation models. The "MACE" label (blue circles) marks the learning curve of the from-scratch MACE model. The points correspond to the percentages of the full dataset, namely 2, 5, 10, 20, 40, 60, 80 and 100 %.

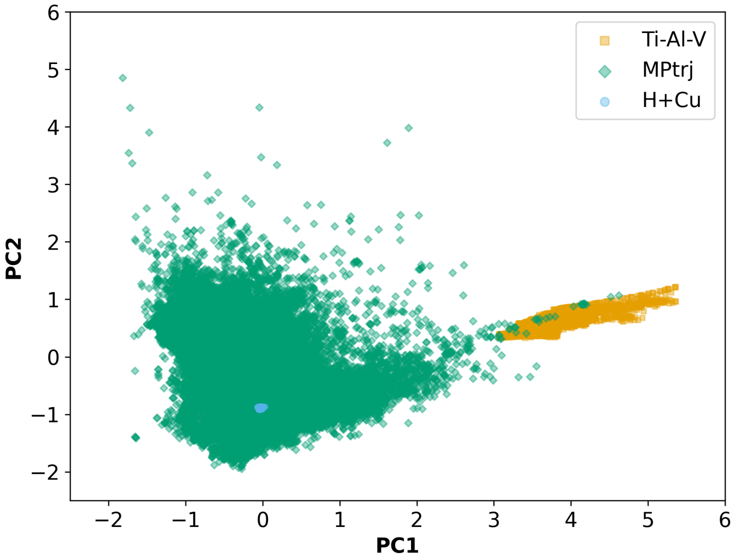


FIG. S13. **Principal Component Analysis (PCA) of the MACE-MP-0 “small” features (invariant descriptors).** Points correspond to configurations from MPtrj (green diamonds), Cu-H (blue circles), Ti-Al-V (yellow squares).

[1] Westermayr, J. & Maurer, R. J. Physically inspired deep learning of molecular excitations and photoemission spectra. *Chem. Sci.* **12**, 10755–10764 (2021).

[2] Schütt, K. T. *et al.* SchNet: A continuous-filter convolutional neural network for modeling quantum interactions (2017). Preprint at https://arxiv.org/abs/1706.08566.

[3] Allen, C. S. & Bartók, A. P. Multi-phase dataset for Ti and Ti-6Al-4V (2025). Preprint at https://arxiv.org/abs/2501.06116.
